# Bite Injuries among Vaccination Staff Participating in a Mass Canine Rabies Vaccination Campaign, Haiti 2016–2017

**DOI:** 10.4269/ajtmh.21-0241

**Published:** 2021-09-07

**Authors:** Rudy T. Kirkhope, Andrew D. Gibson, Pierre Dilius Augustin, Kelly Crowdis, Natael Fénelon, Ewan T. MacLeod, Marco A. N. Vigilato, Emily G. Pieracci, Ryan M. Wallace

**Affiliations:** ^1^ International Animal Health MSc, Biomedical Sciences, College of Medicine and Veterinary Medicine, University of Edinburgh, Edinburgh, United Kingdom;; ^2^ Mission Rabies, Cranborne, Dorset, United Kingdom;; ^3^ The Royal Dick School of Veterinary Studies and the Roslin Institute, University of Edinburgh, Edinburgh, United Kingdom;; ^4^ Department of Animal Health, Ministry of Agriculture, Natural Resources, and Rural Development, Port-au-Prince, Haiti;; ^5^ Christian Veterinary Mission, Port-au-Prince, Haiti;; ^6^ Pan American Health Organization, Port-au-Prince, Haiti;; ^7^ Infection Medicine, Biomedical Sciences, College of Medicine and Veterinary Medicine, University of Edinburgh, Edinburgh, United Kingdom;; ^8^ Pan American Health Organization, Washington, District of Columbia;; ^9^ National Center for Emerging and Zoonotic Infectious Diseases, Centers for Disease Control and Prevention, Atlanta, Georgia

## Abstract

Elimination of dog-transmitted human rabies worldwide will require large-scale dog vaccination campaigns. However, this places participating vaccinators at increased risk. Data from the 2016–2017 Haitian mass rabies vaccination campaign was analyzed to determine dog bite incidence among vaccinators. A survey was then developed for completion by all identifiable bitten vaccinators covering demographics; experience and training; bite episode details; attitudes toward dogs and rabies; and medical care. A parallel group of unbitten vaccinators was also surveyed. Dog bite incidence was 0.03% (43/127,000) of all dogs vaccinated. The capture, vaccinate, and release method of vaccination carried a significantly higher risk of dog bite (0.35%, 6/1,739 vaccinations) than other methods (*P <* 0.001). Twenty-seven bitten vaccinators, and 54 control vaccinators were included in the survey analysis. No differences were found between groups in demographics, experience, or training. However, bitten vaccinators were significantly more likely than the control group to have experienced a dog bite before the study period (*P <* 0.001). This may be associated with a lesser appreciation of dogs, and/or a poorer ability to interpret dog behavioral signals within this group. Although 98% of the control group indicated they would seek medical care for a dog bite, only 35% of bitten vaccinators sought such care. On a yearly basis, for the Haitian campaign, a full series of postexposure rabies vaccinations for all bite victims would prove more cost-effective than preexposure vaccination of all vaccinators. These findings may prove useful for the planning and safety of future mass dog vaccination campaigns.

## INTRODUCTION

Despite recognition of its public health significance by the WHO as early as 1950, rabies remains prevalent throughout most of the world.^1^^,^^2^ Recent studies suggest a global burden of between 1.7 and 3.7 million disability adjusted life years, 26,000 to 159,000 deaths, and 8.6 to 120 billion US dollars yearly.^3^^–^^5^ Although all mammals are susceptible, the most significant reservoir species in relation to public health are those belonging to the family *Canidae*. Consequently, as humans and domestic dogs live in close proximity in most cultures, > 99% of all reported human rabies cases are caused by exposure to an infected dog.^6^ This burden is almost entirely borne by developing countries, where the rate of canine vaccination is low, and life-saving rabies biologics are often unavailable or inaccessible.^6^^,^^7^ In an effort to reduce human and animal suffering as a result of rabies, the WHO, World Organization for Animal Health and the Food and Agriculture Organization of the United Nations have declared a goal to eliminate dog-mediated human rabies deaths by the year 2030 (“Zero by 30”).

Significant progress has been made toward the elimination of dog rabies throughout most of the Western Hemisphere.^8^ However, the incidence in Haiti remains disproportionately high, causing an estimated 130 human deaths, and 18% of canine deaths annually.^4^^,^^9^ Officially reported human cases comprise only a fraction of this number, reflecting the infrastructural challenges of detecting, diagnosing, and reporting suspect cases that are present in many rabies-endemic countries.^10^ As domestic dogs appear to be the only significant reservoir of rabies in the country at present, elimination of dog-mediated human rabies appears achievable in Haiti if dog vaccination programs can be effectively implemented.^8^^,^^11^

Between 2011 and the present day, the US CDC has been supporting the implementation of a rabies surveillance and control program in Haiti in collaboration with the Haitian Ministry of Agriculture, Natural Resources and Rural Development (MARNDR), Ministry of Health (MSPP), Pan American Health Organization (PAHO) and Christian Veterinary Mission (CVM).^12^ Mass vaccination campaigns were extensively evaluated beginning in 2016, and programmatic improvements continue to be implemented in an effort to reach at least 70% coverage of the dog population.^13^ Between 2016 and 2017, approximately 200,000 dogs were vaccinated by multiple field teams throughout Haiti. The use of a smartphone application (Mission Rabies app; Worldwide Veterinary Services) has greatly assisted in objectively ensuring adequate vaccination coverage of the dog population in each community.^14^^,^^15^

As dog vaccination efforts further intensify across the world, striving toward “Zero by 30,” more vaccinators will be needed.^16^ A single vaccinator can encounter thousands of unfamiliar animals during a campaign, each presenting a risk of bite injury and rabies exposure. There is little if any information available as to the incidence of dog bite injury to vaccinators during a mass vaccination campaign, factors that might mitigate this risk, or the cost of bite treatments incurred by the campaign. This study was conducted during a national vaccination campaign in Haiti to ascertain the rate of dog bite injuries to vaccinators, examine the healthcare seeking behavior of bite victims, and investigate risk factors for bite injuries in an effort to develop planning and safety guidelines in the context of future mass dog vaccination campaigns.

## MATERIALS AND METHODS

Data from the Haitian mass dog rabies vaccination campaign between August 2016 and October 2017 was collected using a smart phone application (Mission Rabies app; Worldwide Veterinary Services 2015). Once a vaccination session is launched within the application, vaccination events are continuously uploaded to a central database. Each reported vaccination receives a code, which groups the individual vaccination event to a particular vaccination session, typically encompassing one day of vaccination by one vaccination team. Additional information collected for each vaccination includes a unique identity code, date, project name (region where vaccination is taking place), user ID number, and GPS coordinates. This detailed data is referred to as vaccination point data (VPD). Once vaccination is complete at the end of the working day, the user is taken through a series of follow-up questions before the session is closed out and uploaded, including names of vaccinators, the method of vaccination taking place (central point [CP]; capture, vaccinate, release [CVR]; or door to door [DD]^17^), and whether a bite injury occurred and if so, details of the incident. This data is referred to as the daily vaccination data (DVD). The DVD and VPD contain a linkable identification variable.

### Data analysis.

The VPD and DVD datasets were merged using SAS/STAT^®^ software version 9.3 (SAS institute, Cary, NC) to obtain an accurate vaccination count in association with the additional information. Both VPD and DVD for the vaccination day needed to be present for each data entry to be eligible for inclusion in this study. If DVD and VPD daily vaccination counts differed for a unique entry (around 1% of total merged records), then the higher count was considered the “true count.” The remainder of the data analysis was completed using Microsoft Excel (Version 12.0, 2007).

### Survey preparation.

A written survey was developed to be completed by all identifiable vaccinator bite victims (Supplemental Survey 1). The information requested included basic demographics; experience and training; knowledge and attitudes toward dogs and rabies (using prepared statements and a 7-point rating scale); and details of pre- and postexposure medical care. A control group survey was also developed to be completed by vaccinators that had not been bitten during the period under investigation to identify differences between groups (Supplemental Survey 2). The goal was to obtain 90 completed surveys from control workers (a roughly 1:2 case–control ratio) in an attempt to provide adequate statistical power for multiple case–control risk factor comparisons. Besides information regarding the bite episode, the surveys were identical. The surveys were created in English, then with the assistance of Haitian Creole speakers (Kelly Crowdis, Pierre Augustin, and Frantzot Estime) translated into Creole. Surveys were completed by telephone interview between March and July 2018. Written survey answers were then translated back into English for analysis.

### Statistical analysis.

Descriptive statistics and comparisons between groups was completed using WinPepi “Describe” and “Compare” software (Version 3.77; Abramson 2016). Confidence intervals were set at 95%. Rates and proportions were compared using either Pearson’s χ^2^, or Fisher’s exact two-tailed test for small sample sizes. Numerical observations were compared using Mood’s median test.

## RESULTS

### Data set analysis.

A total of 127,035 unique dog vaccinations, performed by 370 vaccination staff, were included in the datasets spanning the period from August 2016 to October 2017 (Table 1). Of these, 27,569 were completed using CP vaccination method, 97,727 DD, and 1,739 CVR. The average number of dogs vaccinated per team (typically two individuals) per day was 25 but was highly variable (range 0–188). There were 43 bite incidents reported by vaccination personnel, for an overall bite incidence of three per 10,000 vaccinations (0.03%) and 9.7 per 100 vaccinator-years. Of these, seven occurred during CP vaccination (2.5 per 10,000 vaccinations), 30 during DD (3.1 per 10,000 vaccinations), and six during CVR (35 per 10,000 vaccinations) (Table 1). The CVR vaccination method carried a greater than 10-fold higher risk of bite injury than DD or CP (*P <* 0.001). No individual vaccinator incurred more than one bite episode during the study period, resulting in a yearly bite incidence among vaccinators of 11.4%. No specific region was associated with a higher incidence of bite episodes.

**Table 1:** Vaccinations administered, and number and incidence of dog bite injury experienced by workers, separated by vaccination type during the Haitian national dog rabies vaccination campaign between August 2016 and October 2017

	Vaccination type			
	All vaccinations	Central point	Door to door	Capture, vaccinate, and release
Number of vaccinations	127,035	27,569	97,727	1,739
Vaccinations/team/day*	25 (0–188)	13 (0–188)	29 (0–125)	49 (1–103)
Bite number	43	7	30	6
Bite incidence†	0.03%	0.02%	0.03%	0.35%‡

* Median number of vaccinations (range).

† Bite incidence was calculated as the number of bites expressed as a percentage of total vaccinations administered in each group.

‡ Significant difference in bite incidence from other vaccination types (*P* < 0.001).

### Survey analysis.

A total of 27 bite case follow-up surveys were completed, representing a response rate of 63%. Eighty-one control surveys were returned of which 69 were complete. A random number generator was used to assign numbers to each complete control survey, and the first 54 surveys in numerical order were used for statistical analysis to give a 1:2 ratio of bite to control cases.

### Demographics and work experience.

The median age of all vaccinators surveyed was 42 years old (range 22–65 years) (Supplemental Table 1). There was no significant difference in age between those bitten and those not bitten. Females comprised 12.3% of the survey respondents. Although the percentage of females in the bite group (18%) was twice that of the control group (9%), this was not statistically significant (*P =* 0.29). Of the bite group, 25/27 (93%) described their job title as veterinary agent (completed a 12-week certification program in animal health). The remaining two were a veterinarian and a program assistant. Of the control group 38/54 (70%) were veterinary agents. The remainder were assistants (11), telephone operators (3), and a veterinarian. Median dog vaccination experience of all vaccinators was 5 years, with 3 years in the current MARNDR program. However, experience varied greatly from 1 to 32 years. There was no significant difference between groups in terms of their vaccination experience (5 versus 6 years, *P =* 0.53). The median lifetime number of dogs vaccinated per worker was 1,000, also with large variation (100–12,000). There was no difference between groups in lifetime number of dogs vaccinated (950 versus 2,000, *P =* 0.15).

### Preexposure prophylaxis and prior bite experience.

Thirty three percent of all survey respondents reported that they had been vaccinated against rabies (received preexposure prophylaxis [PrEP]) prior to the campaign, with no significant difference noted between bite and control groups (44% versus 22%, *P =* 0.07) (Table 2). All of these vaccinations besides one were obtained after 2013. Eighty-two percent of all respondents reported they had discussed PrEP with a supervisor before starting work, and were informed they would receive the vaccination for free, if desired.

**Table 2:** Comparison of preexposure rabies vaccination prevalence, prior dog bite episode experience, and average number of bites before the current study period between bite and control groups

	Control group	Bite group	*P* value
PrEP rabies vaccination (%)	22.2 (12.0–35.6)	44.4 (25.5–64.7)	0.07
Prior lifetime bite injury (%)	25.9 (15.0–39.6)	74.1 (53.7–88.9)	< 0.001
Among those with a prior bite, persons who sought further medical care (%)	29.4 (13.3–53.1)	38.1 (20.8–59.1)	0.47
Average number of prior bites/worker*	0.39 (0, 0–3)	1.37 (1, 0–8)	< 0.001
Bite incidence/10 Ministry of Agriculture years worked†	0.53	1.61	< 0.001

* Mean value (median, range).

† Bite incidence was calculated as total prior bites reported divided by total years worked for the Ministry of Agriculture (MARNDR).

There was a markedly significant difference between bite and control groups in the proportion of vaccinators reporting a bite injury before the study period, with 74% of the bite group reporting a prior bite, compared with only 26% of the control group (*P <* 0.001). All bite group prior bites, and 17/21 (81%) of control group prior bites were experienced while working on a national rabies vaccination campaign. Bite incidence per 10 MARNDR years worked was also significantly higher in the bite group (1.6) compared with the control group (0.5) (*P <* 0.001). Further medical care was sought in 34% of all prior bite episodes, with no difference found between bite and control groups.

### Attitudes toward dogs and rabies vaccination work.

**Figure 1.**
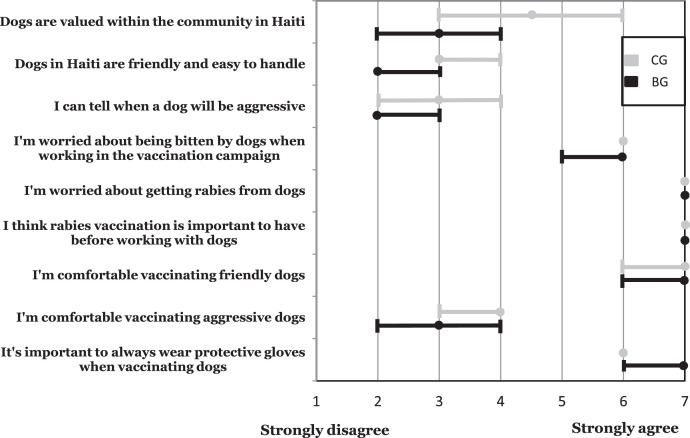
Comparison of attitudes of two groups of Haitian canine rabies vaccination campaign workers toward dogs and rabies vaccination evaluated by their rating of statements on a numbered scale, where a score of 7 indicates strong agreement with the statement, and a score of 1 strong disagreement. Bite group (BG) had experienced a dog bite injury between August 2016 and October 2017 while working in the campaign. Control group (CG) had not experienced a dog bite injury during the same time period. Solid circles indicate median score; bars indicate 96% CI.

Survey participants scored prepared statements dictated to them on a 7-point rating scale, from 1 (strongly disagree) to 7 (strongly agree). Median scores with 96% CI for each statement are illustrated in Figure 1. Both groups agreed or strongly agreed with the statements: “I’m worried about being bitten by dogs while working on the vaccination campaign”; “I’m worried about getting rabies from dogs”; “I think rabies vaccination is important to have before working with dogs”; “I’m comfortable vaccinating friendly dogs” and “It’s important to always wear protective gloves when vaccinating dogs.” The bite group tended toward greater disagreement for the statements: “Dogs are valued within the community in Haiti”; “Dogs in Haiti are friendly and easy to handle”; “I can tell when a dog will be aggressive” and “I’m comfortable vaccinating aggressive dogs.” However, there was no significant difference between groups when confidence intervals were included.

### Vaccinator training.

Almost all vaccinators (80–100%) reported being trained in vaccination technique, bite avoidance, and dog handling (Supplemental Figure 1). However, only 40–60% reported receiving training on the use of hoop nets, control poles, and bite-proof gloves. Of those stating they would have liked further training, the use of the above equipment was requested most frequently. There were no significant differences between bite and control groups for any of the training categories evaluated.

### Ancillary safety equipment availability and usage.

All vaccination teams routinely carry vaccines, syringes and needles, a cool box, and a sharps container. Ancillary safety equipment was reportedly carried much less frequently, most frequent being a catch pole (36% of respondents), a hoop net (34%) and latex gloves (24%) (Supplemental Table 2). When available, latex gloves were reportedly used routinely, or during the bite incident by 83% of vaccinators. Meanwhile, bite-proof gloves, catch poles, hoop nets, syringe poles, and leashes were reported as routinely used, or used during the bite incident by 20–65% of the vaccinators who carried them, with no difference between bite and control groups. No other equipment was reported as being routinely carried by vaccinators.

### Bite episode details (Bite Group).

The majority of biting dogs (85%) were either known to be, or considered to be owned. Bite victims reported that they were familiar with the biting dog or the dog’s owners in 26% of cases. Vaccination method being undertaken at the time of bite in the surveyed workers was either DD exclusively (53%), or CP combined with DD (47%). All but one of the bite victims were performing the role of vaccinator when bitten. The remaining victim was performing the role of assistant. The most common times for experiencing a bite were either during capture/restraint of the dog (26%) or during administration of the vaccine (52%). Anatomical location of the bite was variable, but most frequently on either the hand (38% of all bites), foot (23%), or finger (19%) (Supplemental Figure 2). The majority of bites were deep enough to cause bleeding (89%).

### Proposed versus actual response in the case of a bite episode.

Table 3 compares control group responses regarding a hypothetical bite, with the actual course of action bite victims took at the time of their bite. One of the bite group surveys was incomplete for this section, so a total of 54 control, and 26 bite group surveys were included in final analysis.

**Table 3:** Proposed (control group) and actual (bite victim group) behavior of Haitian dog vaccination workers after experiencing a dog bite

Control group					Bite group				
If you were bitten during your work on the vaccination campaign, what should happen to the dog?	Monitor at home			48/54 (88.9)	What happened to the dog that bit you?	Monitored at home (healthy after 14 days)			17/26 (65)
	Euthanize			5/54 (9.2)		Euthanized			0/26 (0)
	Don’t know			1/54 (1.8)		Don’t know			9/26 (35)
If you were bitten, would you report the bite to someone on your vaccination team?*	Yes	52/54 (96.3)	Vaccination partner	43/52 (82.7)	Did you report the bite to someone on your vaccination team?*	Yes	26/26 (100)	Vaccination partner	25/26 (96.1)
			Another vaccinator	38/52 (73.1)				Another vaccinator	26/26 (100)
			Local coordinator	45/52 (86.5)				Local coordinator	22/26 (84.6)
			National coordinator	11/52 (21.1)				National coordinator	4/26 (15.4)
	No			2/54 (3.7)		No			0/26 (0)
If you were bitten during the vaccination program, what would you do?*	Nothing/Go home			0/54 (0)	After you were bitten, did you wash the wound?	No			1/26 (3.8)
	Wash wound with water only			3/54 (9.2)		Yes	25/26 (100)	Yes with water only	0/25 (0)
	Wash wound with soap and water			49/54 (87.0)				Yes with soap and water	24/25 (96.0)
	Wash wound with antiseptic			17/54 (25.9)				Yes with antiseptic	1/25 (4.0)
	Seek medical care from a healthcare facility			53/54† (98.1)	Did you seek further medical attention?	Yes			9/26† (34.6)
	Seek medical care from a veterinarian			1/54 (1.8)		No			17/26 (65.4)
	Seek care from a priest			3/54 (5.5)	If yes, what did medical care consist of?*	Wound washing			0/9 (0)
	Seek care from a friend			7/54 (13.0)		Wound disinfection			4/9 (44.4)
						Tetanus antitoxin			4/9 (44.4)
						Bandage			1/9 (11.1)
						Antibiotics			2/9 (22.2)
	Seek rabies vaccination			45/54 (83.3)		Rabies vaccination			3/9 (33.3)
	Seek rabies immunoglobulin			13/43 (30.2)		Rabies immunoglobulin			0/9 (0)

Results are presented as a proportion, and as a percentage (%) of all completed responses.

* Multiple responses permitted.

† Significant difference between control and bite groups (*P* < 0.001).

If bitten by a dog during the vaccination campaign, 89% of the control group were aware that the correct plan of action for the biting dog was home monitoring in case signs of rabies developed. However, while 17/26 (65%) of the bite group reported that the biting dog was indeed monitored at home (and healthy after 14 days), 35% were unaware of what happened to the animal. When asked about medical care following a bite, 87% of control group workers indicated they would wash the wound with soap and water, 98% indicated they would seek medical care from a healthcare facility, and 83% indicated they would seek rabies vaccination. In the case of an actual bite, 24/26 (92%) of the bite group reported they washed the wound with soap and water, but only 9/26 (35%) ultimately sought further medical care from a healthcare facility, significantly different from the control group response (*P <* 0.001). Unfortunately, only three individuals responded as to why further medical care was not sought. All three stated that their bite wound was “not serious” or “only a scratch.” However, two out of these three victims had previously reported that the bite resulted in bleeding.

Of the 17 bite group individuals who were aware the biting dog remained healthy, 12/17 (70%) did not seek further medical care. Meanwhile 5/9 (55%) of the bite group who were unaware of the biting dog’s health status also did not seek further medical care, suggesting knowledge of the dog’s health status did not affect healthcare seeking behavior (*P =* 0.67). Of the nine individuals seeking further medical care, only three reported receiving a rabies vaccine at the healthcare facility. Other treatments reported were tetanus antitoxin, antibiotics, and bandaging. One responder specifically mentioned that they were told there was no rabies vaccine available at that facility. When medical care was sought, the reported average time to obtaining this care was 1 day (range 2 hours to 3 days). The three bite victims who received rabies vaccine were treated within 2 days of the bite, and in all of these cases the dog was monitored and known to be healthy at 14 days. Although not officially recorded in the survey, no cases of canine or human rabies were associated with the investigated bite incidents.

### Bite avoidance.

Bite victims believed their bite could have been avoided in 60% of cases. Supplemental Figure 3 compares suggested solutions that would have prevented the bite investigated here (bite group) or would prevent bites in general (control group). Both groups strongly agreed that better training would be/would have been of assistance in preventing bites (87% of all respondents). A significantly greater proportion of control group than bite group (93% versus 69%) believed that better control by owners of their dogs would prevent/would have prevented bite injury (*P <* 0.05). Meanwhile, a significantly greater proportion of bite group than control group believed that the use of bite-proof gloves (77% versus 26%) (*P <* 0.001) would prevent/would have prevented a bite injury. Both groups (71% of all respondents) tended to believe that additional equipment besides bite-proof gloves would prevent/would have prevented bite injury. The most common items cited were a hoop net, rabies pole, leash, and muzzle. Eighty-eight percent of bite victims reported that they had not changed their work methods significantly since the bite.

## DISCUSSION

### Bite incidence.

During the vaccination of over 127,000 dogs by an experienced workforce of 370 vaccination personnel, 43 staff members experienced bite injuries. Haiti’s 2016/2017 vaccination campaign spanned a roughly 12-month period, so approximately 500 dogs were vaccinated on each working day. Therefore, data from this survey would suggest that similarly organized campaigns would expect an average of one bite injury each week, or 50 bite injuries per campaign-year.

The bite incidence using the CVR method of vaccination was significantly higher (around 10 times the risk) than the other methods. The sample size for this method was relatively small compared with the other methods, as CVR was only operated in select locations. Given the increased likelihood of roaming dogs being aggressive when approached, this is not an unexpected finding. This finding may prove insightful for countries such as India where CVR vaccination is frequently used.^14^^,^^18^ Thorough vaccinator training, ancillary equipment, and PrEP of vaccinators would appear prudent before commencing a CVR campaign. Unfortunately, there were no bite case surveys completed by vaccinators performing CVR vaccination at the time of their bite, so this may affect the relevance of the survey findings in regards to CVR campaigns.

### High-risk workers.

A recent large-scale survey of Haitian community members found a dog bite incidence of 0.9 bites per 100 life years.^19^ Unsurprisingly, Haitian vaccinators had a bite incidence over five times greater than that of community members, supporting an increased risk of dog bite with this occupation. However, the dog bite incidence among bitten vaccinators in this study was 15 times higher than that of community members, suggesting that a subset of vaccinators are more prone to experience a dog bite during performance of their work regardless of age, experience, and the availability or use of safety equipment.

Although not statistically significant, the bite group’s tendency toward greater disagreement with the statements: “Dogs are valued within the community in Haiti”; “Dogs in Haiti are friendly and easy to handle”; “I can tell when a dog will be aggressive”; and “I’m comfortable vaccinating aggressive dogs” may indicate a lesser appreciation of dogs, and/or a poorer ability to interpret dog behavioral signals within this higher risk group. Additionally, of bite group vaccinators who were carrying ancillary safety equipment, less than half were using it at the time of their bite, supporting the theory that this group may have difficulties identifying which dogs pose a significant threat of biting. Identification of persons with a history of dog bites, and additional training for these individuals, alongside vaccinators that are bitten during the campaign may help reduce future injuries. It would seem prudent to include recognition of dog behavioral signals as part of basic vaccinator training.^20^

### Healthcare seeking behavior, preexposure and postexposure prophylaxis.

The proportion of around one-third of vaccinator bite victims seeking further medical care corresponds with two prior studies of the general public in Haiti.^19^^,^^21^ Given the relatively low adoption of voluntary PrEP by vaccinators, this continues to raise concerns regarding adequate rabies prophylaxis. Haiti has an estimated 700,000 dogs, and Haiti’s vaccination program is intending to scale-up operations to be able to vaccinate at least 500,000 dogs every year. For a scaled-up campaign, if 1,500 vaccinators received mandatory two-dose PrEP at an average cost of $40, this would result in a campaign cost for PrEP of approximately $60,000. In comparison, if bite rates among vaccinators remain similar to what was observed in this study, an estimated 150 bite injuries would occur per campaign-year. At an estimated cost of $126 per four-dose postexposure prophylaxis (PEP) series, this would incur a campaign cost for PEP of $18,900.^22^ Assuming a standard vaccination cost of $2.18 per dog, these PrEP and PEP costs represent 5.5% and 1.7% of the total campaign budget, respectively.^23^ Consideration as to whether these are one-time costs or annual is difficult, since staff turnover and repeat bites would alter costs substantially. Presumably, each scenario would continue to encounter some ongoing annual costs for human rabies vaccination.

The cost for PEP could be reduced further if it was targeted only to those individuals bitten by dogs whose rabies-infection status could not be confirmed through diagnosis or at-home observation. Since 2013, Haiti has been developing integrated bite case management (IBCM) and animal rabies surveillance (HARSP) programs to identify rabid dogs as quickly as possible, and be able to safely withhold PEP provision in most cases.^19^^,^^24^^,^^25^ Therefore, specifically in the context of the Haitian vaccination campaign and HARSP, mandatory PrEP would not result in significant health benefits in comparison to costs, so long as all bitten vaccinators were identified and dogs adequately assessed. The case for mandatory PrEP for vaccinators remains stronger for campaigns where there are no animal surveillance programs yet in place, and those using the CVR method of vaccination.^14^^,^^26^^,^^27^

### Bite avoidance strategies.

Equipment designed for handling fractious animals could potentially reduce bite incidence. However, this study demonstrated that even when available, additional equipment was only intermittently used. Consistent usage of such equipment increases the time spent vaccinating each animal, and increases costs through equipment purchase and longer campaign durations. Increasing vaccinators’ familiarity with catch poles, nets, leashes, and muzzles through greater training opportunities, and then increasing the availability of equipment in select locations should still be considered to evaluate whether more widespread provision would prove socioeconomically beneficial.

The majority of bite group vaccinators did believe that bite-proof gloves would have prevented their bite injuries, a belief supported by the high percentage of bites incurred on the hand or finger (57% of all bites). However, when actually available, both groups reported using bite-proof gloves less than 20% of the time. Although effective against bites, these gloves are cumbersome, and severely decrease manual dexterity when handling a syringe. They are also expensive (typically > $100 per pair). Although less protective against a bite, work gloves (i.e., gardening gloves) cost less than $15, and would likely confer protection against minor bites and scratches. Of additional note, the second most frequent bite location reported by bite victims was on the foot (23% of all bites). Further program evaluation should be conducted to ascertain the acceptance and usefulness of work gloves and sturdy footwear in reducing vaccinator bite incidence and severity.

### Potential for recall bias.

The average time between bite incidence and survey completion was around 1 year, with a range of 5–16 months. Training and opinion-based survey responses would be unlikely to have changed over this time period. Recollection of safety equipment used at the time of the bite, bite episode details, and medical care sought/provided may have diminished over time. However for the most part intricate details were not requested, thus minimizing the potential for recall bias in survey participants.

## CONCLUSION

To the authors’ knowledge, this is the first study to determine an incidence rate for dog bites in a population of vaccinators participating in a mass dog vaccination campaign. It also identified some potential risk factors for bite injuries. Even among this trained and experienced vaccinator workforce, dog bites were relatively frequent. As mass vaccination increases globally in conjunction with the goal of eliminating dog-mediated human rabies deaths, this information should prove useful in the planning of future campaigns from an occupational health and socioeconomic viewpoint.

## Supplemental Material


Supplemental materials
