# Supplementary material for: Bite Injuries among Vaccination Staff Participating in a Mass Canine Rabies Vaccination Campaign, Haiti 2016–2017
Source: Am J Trop Med Hyg. 2021 Sep 7;105(6):1582–9. doi: 10.4269/ajtmh.21-0241 (PMC8641350; doi:10.4269/ajtmh.21-0241)
Supplement: Supplementary file 1 [file tpmd210241.SD1.pdf]

Supplemental Materials

		Control group	Bite group
**Age (years)[a]**		42 (22 - 65)	43 (24 - 61)
**Sex (%)**	**M**	90.7	81.5
	**F**	9.3	18.5
**Experience[a] (years)**	**Non-MARNDR**	5 (1 - 25)	6 (1 - 32)
	**MARNDR**	3 (1 - 23)	6 (1 - 32)
**Average career number of dogs vaccinated[a]**		935 (100 - 12000)	2000 (300 - 12000)

Table S1: *Demographics and work experience of surveyed workers participating in the Haitian dog rabies vaccination campaign between August 2016 and October 2017.*

[a]*Median value (range)*

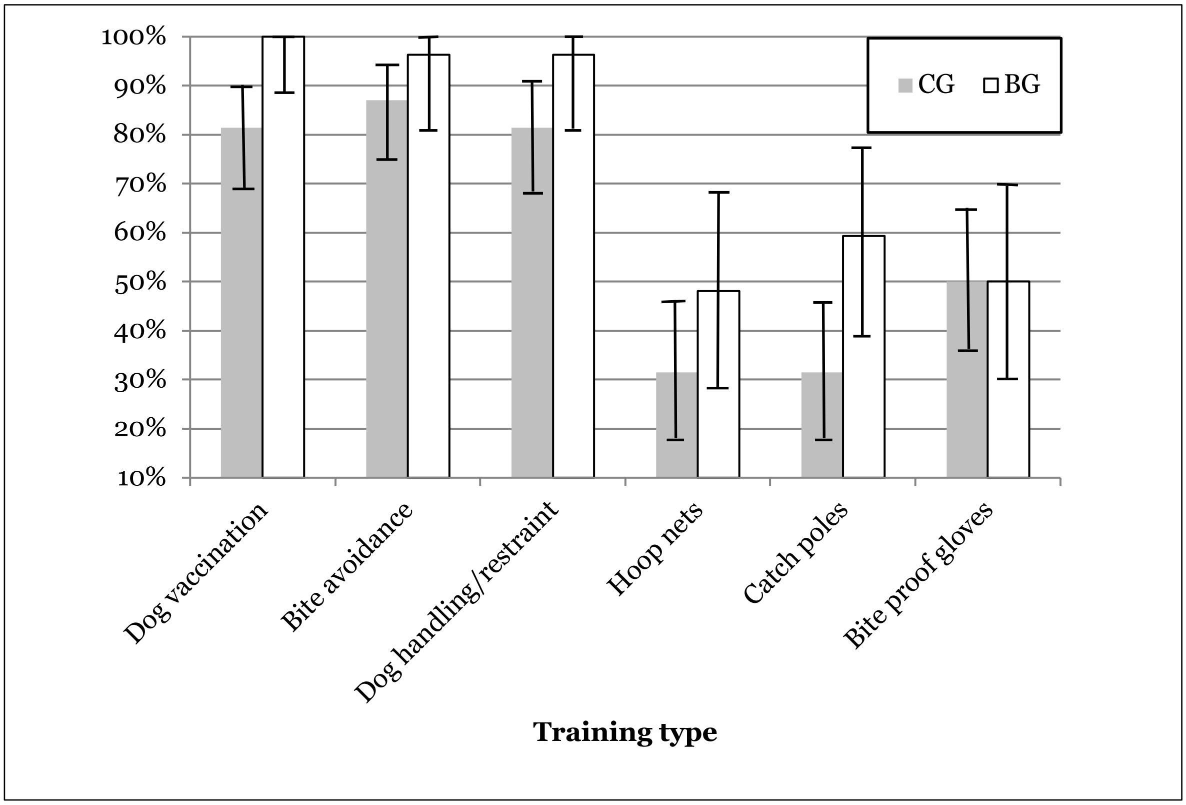

Figure S1: *Percentage of surveyed workers participating in the Haitian dog rabies vaccination campaign between August 2016 and October 2017 who indicated they had received specific types of training prior to commencing work. CG: Control group. BG: Bite group.*

	**Control Group**		**Bite Group**	
**Equipment**	Available	Used routinely	Available	Used in bite episode
Latex gloves	*29.6 (23.6-51.9)*	*24.1 (13.5-37.6)*	*18.5 (6.3-38.1)*	*18.5 (6.3-38.1)*
Work gloves	*0 (0-6.6)*	*0 (0-6.6)*	*0 (0-12.8)*	*0 (0-12.8)*
Bite proof gloves	*7.4 (2.1-17.9)*	*1.8 (0-9.9)*	*3.7 (0-19.0)*	*0 (0-12.8)*
Catch pole	*35.2 (22.7-49.4)*	*11.1 (4.2-22.6)*	*37.0 (19.4-57.6)*	*7.4 (1.0-24.3)*
Hoop net	*31.5 (19.5-45.5)*	*11.1 (4.2-22.6)*	*37.0 (19.4-45.5)*	*11.1 (2.3-29.1)*
Syringe pole	*5.6 (1.2-15.4)*	*1.8 (0-9.9)*	*0 (0-12.8)*	*0 (0-12.8)*
Leash	*22.2 (12.0-35.6)*	*13.0 (5.4-24.9)*	*11.1 (2.3-29.1)*	*7.4 (1.0-24.3)*

Table S2: *Percentage (95% confidence interval) of vaccinators surveyed who reported ancillary equipment as available, and if available routinely used during the vaccination campaign (Control group), compared to workers who had experienced a bite episode and reported equipment available, and if available used at the time of their bite (Bite group).*

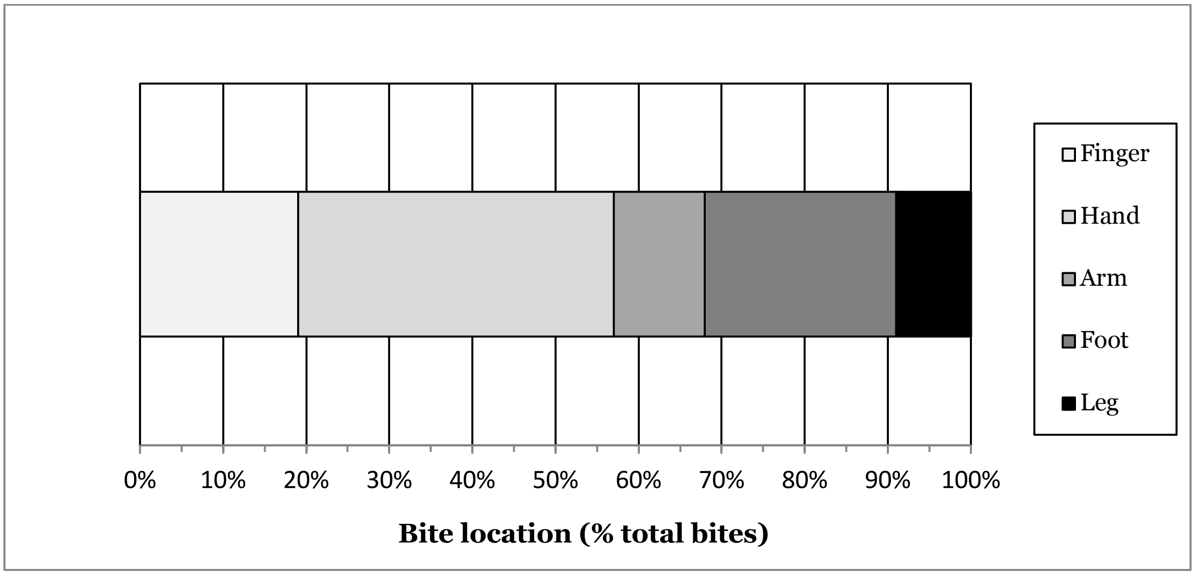

Figure S2: *Anatomical location of bite injuries sustained by the bite group, presented as a percentage of total bite injuries (n= 26).*

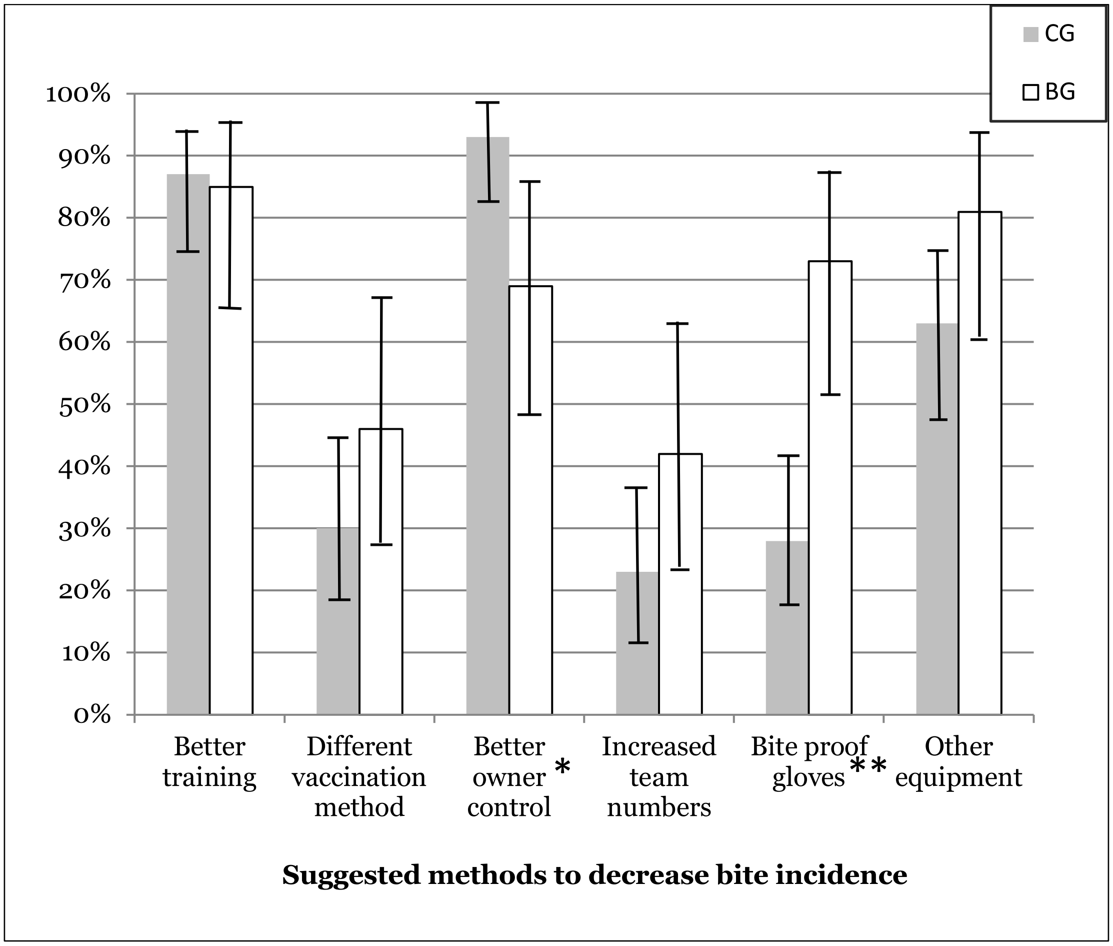

Figure S3: *Percentage of surveyed workers participating in the Haitian dog rabies vaccination campaign between August 2016 and October 2017 who agreed that each suggested method would decrease bite incidence during vaccination campaigns. CG: Control group. BG: Bite group.*

** Significant difference between control and bite groups (P < 0.05)*

*** Significant difference between control and bite groups (P < 0.001)*

Survey S1

Vaccination Campaign (Bite Victim) Survey

BASIC INFORMATION

1) **Name**:______________________________

2) Sex

M □ F □

3) Age _____

4) What commune do you live in? __________________________________

5) What sectional communal do you live in? _____________________________

6) What is/was your role in the rabies vaccination programme?

Veterinarian □ Veterinary agent □ Administration □ Owner □ Assistant □ Announcer □ Other □

7) On a scale of 1 – 7, with 7 being "Strong Yes" and 1 being "Strong No", please tell us your feelings about these statements:

a. Dogs are valued within the community in Haiti □

b. Dogs in Haiti are friendly and easy to handle □

c. I can tell when a dog will be aggressive □

d. I'm worried about being bitten by dogs when working in the vaccination campaign □

e. I am worried about getting rabies from dogs □

f. I think rabies vaccination is important to have before working with dogs □

g. I am comfortable vaccinating friendly dogs □

h. I am comfortable vaccinating aggressive dogs □

j. It is important to always wear protective gloves when vaccinating dogs □

EXPERIENCE

8) How many years have you been vaccinating dogs in Haiti?

______ years

9) How many years have you participated in the MARNDR national rabies vaccination programme?

______ years

10) Approximately how many dogs have you vaccinated in your lifetime?

_____ dogs

11) Have you received any of the following training?

1 – Training in vaccinating dogs against rabies **Y / N / Unknown**

2 – Training in bite avoidance or prevention **Y / N / Unknown**

3 – Training in dog handling and restraint **Y / N / Unknown**

4 – Training with hoop nets **Y / N / Unknown**

5 – Training with catch poles **Y / N / Unknown**

6 – Training with bite-proof gloves **Y / N / Unknown**

7 – Is there any training you would have liked to have, but did not receive prior to the vaccination campaign this year?

Please explain:____

RABIES PROPHYLAXIS

12) Have you ever received pre-exposure rabies vaccination (vaccination against rabies to protect in case of a bite)? Y □ N □ Don't know □

If yes:

In what years did you receive vaccination? _____________________

Who paid for the vaccine? ____________________

Where did you get the vaccine from?_______________________

13) Before you started work on the campaign *this year*, did a vaccination coordinator discuss you getting vaccinated to protect against rabies?

Y □ N □ Don't know □

If yes, what did they recommend?

You should be vaccinated and it will be free □

You should be vaccinated, but it will be at your own expense □

It is not necessary □

14) If you were vaccinated just before starting ***this year's*** **campaign:**

Who paid for the vaccine?________________________________

Where did you get the vaccine from?___________________________

15) Have you been bitten by a dog before the bite you experienced this year?

Y □ N □

If yes, on how many occasions in the past have you been bitten by a dog _____

If yes, was this while working in a vaccination campaign? For bite #1: Y □ N □

For bite #2: Y □ N □

For bite #3: Y □ N □

If yes, did you seek medical care for the bite? For bite #1: Y □ N □

Fr bite #2: Y □ N □

For bite #3: Y □ N □

BITE EPISODE DETAILS

16) In *this year's* bite episode, was the dog that bit you

Your dog □

A dog you knew (but not yours) □

A dog you did not know (but probably owned) □

A dog you did not know (but probably not owned) □

Don't know □

17) What type of vaccination were you performing?

Fixed point □

Door to door □

Capture vaccinate and release □

18) What role were you performing when bitten?

Vaccinator □

Assistant □

Sensitization □

19) What equipment did you have with you the day you were bitten. Please indicate if you were using the equipment at the time of the bite:

1 – Latex gloves	Available: Y / N / Unknown	Used: Y / N / Unknown
2 – Work gloves	Available: Y / N / Unknown	Used: Y / N / Unknown
3 – Bite proof gloves	Available: Y / N / Unknown	Used: Y / N / Unknown
4 – Catch pole (lasso)	Available: Y / N / Unknown	Used: Y / N / Unknown
5 – Hoop net	Available: Y / N / Unknown	Used: Y / N / Unknown
6 – Syringe pole	Available: Y / N / Unknown	Used: Y / N / Unknown
7 – Leash	Available: Y / N / Unknown	Used: Y / N / Unknown

8 – Any other equipment? Available: Y / N / Unknown Used: Y / N / Unknown

Please explain____

20) During what part of the procedure did the bite occur?

Before handling the dog □

During capture □

While an owner was restraining □

While you were restraining □

During administration of vaccine □

After vaccination □

21) Where were you bitten? (*check all that apply*):

Finger □ Hand □ Arm □ Leg □ Foot □ Head/Face □ Torso □ Neck □

Describe the bite in more detail:____

22) How serious was the bite?

Superficial wound, no blood □

Puncture wound with blood □

Deep puncture wound with blood□

Multiple puncture wounds with blood □

23) What happened to the dog?

Monitored at home □

Euthanized □

Don't know □

If the dog was monitored, was it healthy after 14 days?

Yes □

No, but it was alive □

No, it died □

Don't know □

24) Did you report the bite on the Mobile Phone Survey?

Y □ N □

25) Did you report the bite to someone on your vaccination team?

Y □ N □

If yes, to whom did you report the bite?

Vaccination partner □

Another vaccinator □

Local coordinator □

National coordinator □

Other □

MEDICAL TREATMENT

26) Did you wash the wound?

No □

Yes, with water only □

Yes, with soap and water □

Yes, with antiseptic □

If not, why not? ____

27) Did you seek further medical attention?

Y □ N □

If not, why not? ________________________________

28) If medical care was sought, how soon after the bite?

Time in minutes, hours or days ____ minutes ______ hours ______days

29) What did care consist of? (check all that apply):

i. Wound washing □
ii. Wound disinfection □
iii. Tetanus antitoxin
iv. Antibiotics □
v. Bandage □
vi. 1 Rabies vaccination □
vii. 2-3 Rabies vaccines □
viii. 4-5 Rabies vaccines □
ix. Rabies immunoglobulin □

30) Who recommended this care?

Vaccination coordinator □

Healthcare provider □

Friend □

Family □

I decided myself □

31) If you consulted a health care provider, was this the care they recommended?

Y □ N □

32) If you didn't follow the recommendations of the health care provider, why not?

Treatment not available □

Healthcare facility too far away □

Too expensive □

Not considered important enough □

Other (explain) ____________________________________

33) Did you seek care from any other sources?

Traditional healer ☐

Priest ☐

Veterinarian ☐

Family member ☐

Other ☐ Who?____________________________

34) Did you miss any time from the vaccination campaign due to the bite?

Y ☐ N ☐

If yes, how much time did you miss?___________

PREVENTION

35) Do you think the bite could have been avoided?

Y ☐ N ☐

If yes, how could the bite have been avoided?____

36) Do you think any of the following would have helped prevent your bite episode in this vaccination campaign? (*ask all, check all that apply*)

Better training before starting work ☐ What training?______________________

Different vaccination method ☐ What method? ______________________

Better owner control of their dog ☐ How?_________________________

Increase vaccination team numbers ☐ How many and why?______________________

Bite proof gloves ☐

Other equipment? ☐ What equipment and why?__________________

Other? ☐ Explain _______________________________

37) Have you changed your work methods after your bite episode to try to reduce the chances of being bitten again? Y ☐ N ☐

If yes, describe what you changed ____

END

Survey S2

Vaccination Campaign (Control) Survey

BASIC INFORMATION

1) **Name:**______________________________

2) Sex

M □ F □

3) Age _____

4) What commune do you live in? _________________________________

5) What sectional communal do you live in? _____________________________

6) What is/was your role in the rabies vaccination programme?

Veterinarian □ Veterinary agent □ Administration □ Owner □ Assistant □ Announcer □ Other □

7) On a scale of 1 – 7, with 7 being "Strong Yes" and 1 being "Strong No", please tell us your feelings about these statements:

a. Dogs are valued within the community in Haiti □

b. Dogs in Haiti are friendly and easy to handle □

c. I can tell when a dog will be aggressive □

d. I'm worried about being bitten by dogs when working in the vaccination campaign □

e. I am worried about getting rabies from dogs □

f. I think rabies vaccination is important to have before working with dogs □

g. I am comfortable vaccinating friendly dogs □

h. I am comfortable vaccinating aggressive dogs □

j. It is important to always wear protective gloves when vaccinating dogs □

EXPERIENCE

8) How many years have you been vaccinating dogs in Haiti?

______ years

9) How many years have you participated in the MARNDR national rabies vaccination programme?

______ years

10) Approximately how many dogs have you vaccinated in your lifetime?

_____ dogs

11) Have you received any of the following training?

1 – Training in vaccinating dogs against rabies Y / N / Unknown

2 – Training in bite avoidance or prevention Y / N / Unknown

3 – Training in dog handling and restraint Y / N / Unknown

4 – Training with hoop nets Y / N / Unknown

5 – Training with catch poles Y / N / Unknown

6 – Training with bite-proof gloves Y / N / Unknown

7 – Is there any training you would have liked to have, but did not receive prior to the vaccination campaign this year?

Please explain:____

RABIES PROPHYLAXIS

12) Have you ever received pre-exposure rabies vaccination (vaccination against rabies to protect in case of a bite)? Y □ N □ Don't know □

If yes:

In what years did you receive vaccination? ____________________

Who paid for the vaccine? ___________________

Where did you get the vaccine from?______________________

13) Before you started work on the campaign *this year*, did a vaccination coordinator discuss you getting vaccinated to protect against rabies?

Y □ N □ Don't know □

If yes, what did they recommend?

You should be vaccinated and it will be free □

You should be vaccinated, but it will be at your own expense □

It is not necessary □

14) If you were vaccinated just before starting *this year's* campaign:

Who paid for the vaccine?________________________________

Where did you get the vaccine from?___________________________

15) Have you been bitten by a dog before?

Y □ N □

If yes, on how many occasions in the past have you been bitten by a dog _____

If yes, was this while working in a vaccination campaign? For bite #1: Y □ N □

For bite #2: Y □ N □

For bite #3: Y □ N □

If yes, did you seek medical care for the bite? For bite #1: Y □ N □

For bite #2: Y □ N □

For bite #3: Y □ N □

16) Which of the following equipment do you have available to you during the vaccination campaign, and do you routinely use it when vaccinating dogs?

1 – Latex gloves	Available: Y / N / Unknown	Used: Y / N / Unknown
2 – Work gloves	Available: Y / N / Unknown	Used: Y / N / Unknown
3 – Bite proof gloves	Available: Y / N / Unknown	Used: Y / N / Unknown
4 – Catch pole (lasso)	Available: Y / N / Unknown	Used: Y / N / Unknown
5 – Hoop net	Available: Y / N / Unknown	Used: Y / N / Unknown
6 – Syringe pole	Available: Y / N / Unknown	Used: Y / N / Unknown
7- Leash	Available: Y / N / Unknown	Used: Y / N / Unknown
7 – Any other equipment?	Available: Y / N / Unknown	Used: Y / N / Unknown

Please explain____

17) If a dog were to bite you during your work on the vaccination campaign, what should happen to the dog? (*Allow respondent to answer first, and mark what they say. Then read all the suggested responses below and double-mark what they then say*).

	**Initial answer (*no prompts*)**	**Final answer (*after suggesting responses*)**
Monitored at home	☐	☐
Euthanized	☐	☐
Don't know	☐	☐

18) If you were bitten by a dog during the vaccination campaign, would you report it on the Mobile Phone App?

Y ☐ N ☐

19) If you were bitten by a dog during the vaccination campaign, would you report it to someone on your vaccination team?

Y ☐ N ☐

If yes, to whom would you report the bite?

Vaccination partner ☐

Another vaccinator ☐

Local coordinator ☐

National coordinator ☐

Other ◻ Who?________________________________

20) If you were bitten by a dog during vaccination, what would you do? (*Check all that apply. Allow respondent to answer first, and mark what they say. Then read all the suggested responses below and double-mark what they then say*).

		Initial answer (*no prompts*)	Final answer (*after suggesting responses*)
i.	Nothing	◻	◻
ii.	Wash the wound with water only	◻	◻
iii.	Wash the wound with soap and water	◻	◻
iv.	Wash the wound with antiseptic	◻	◻
v.	Seek medical care from a healthcare facility	◻	◻
vi.	Seek medical care from a veterinarian	◻	◻
vii.	Go home	◻	◻
viii.	Seek care from a priest	◻	◻
ix.	Seek care from a friend	◻	◻
x.	Seek rabies vaccination	◻	◻
xi.	Seek rabies immunoglobulin	◻	◻
xii.	Other	◻ What?_____________________	

21) Do you have any suggestions for how bites could be avoided during vaccination campaigns?

Y ☐ N ☐

If yes, how could bites be avoided?____

22) Do you think any of the following would help prevent bites during the vaccination campaign? (*ask all, check all that apply)*

Better training before starting work ☐ What training?_______________________

Different vaccination method ☐ What method? ________________________

Better owner control of their dog ☐ How?__________________________

Increase vaccination team numbers ☐ How many and why?_______________________

Bite proof gloves ☐

Other equipment? ☐ What equipment and why?___________________

Other? ☐ Explain _________________________________

23) Have you changed your work methods during the course of *this year's* vaccination campaign to try to reduce the chances of being bitten? Y ☐ N ☐

If yes, describe what you changed ____

END
